# Molecular Characterization of *Molluscum Contagiosum* Virus: Identification of a Putative New Clade Through *MC021L* Gene Analysis in Diyala Province, Iraq

**DOI:** 10.1155/av/1574406

**Published:** 2025-08-05

**Authors:** Sameer M. Dheyab, Zahraa J. Jameel, Ansam Dawod Salman, Mohammed Kadhom

**Affiliations:** ^1^Department of Biology, College of Science, Diyala University, Baqubah, Diyala 32001, Iraq; ^2^Department of Environmental Health Science, College of Energy and Environmental Science, Al-Karkh University of Science, Baghdad 10081, Iraq

**Keywords:** Diyala Province, *MC021L* gene, *Molluscum contagiosum*

## Abstract

This research concentrated on the molecular diagnosis of *Molluscum contagiosum* (MC) in clinically suspected individuals from Diyala Province, Iraq, through the identification of the *MC021L* gene in skin tissue samples between October 2020 and April 2021. A total of 101 skin lesion samples were collected from patients suspected of MC infection, ranging in age from 5 months to 60 years and including both males and females. Samples were gathered from Baquba Teaching Hospital, the dermatology clinic, and multiple basic healthcare centers, where a tailored questionnaire was developed to capture sociodemographic and clinical details. All samples were analyzed using conventional PCR to detect the *MC021L* gene with specific primers, followed by gene sequencing and phylogenetic analysis. Data were analyzed using SPSS Version 27, with statistical significance set at *p* < 0.05. PCR results showed that 19 samples (18.8%) tested positive, with distinct bands at 979 bp. Sequencing of the *MC021L* locus uncovered 52 nucleic acid variations that are present in almost all samples (including missense mutations in the *MC021L*-encoded glycoprotein). These variations were uniformly distributed across the *MC021L* gene in the viral samples. Phylogenetic analysis revealed that all viral strains belonged to a putative new clade that was located between Subtypes-I and -II. Four isolates were submitted to the NCBI database and assigned the accession numbers LC629162, LC629163, LC629164, and LC629165. The unique genetic sequences of these isolates of the *MC021L* gene confirmed that they do not match any previously identified subtypes, paving the way for further scientific exploration.

## 1. Introduction

*Molluscum contagiosum* (MC), often termed water warts, is a viral dermatological infection characterized by small, dome-shaped pink lesions featuring a central indentation. It is a prevalent condition affecting approximately 122 million individuals, constituting 1.8% of the global population. MC virus (MCV), a dsDNA virus within the Poxviridae family [1], has become the most significant poxvirus affecting humans since the eradication of smallpox. It causes MC, also known as pearl disease [2]. MCV is divided into four genotypes: MCV-1, MCV-2, MCV-3, and MCV-4 [3]. MCV-1 is the main cause of MC in children [4], whereas MCV-2 is sexually transmitted and primarily impacts teenagers and adults [5]. Among its defining traits, MCV promotes infection with little to no inflammation, specifically targets keratinocytes, and employs immune evasion strategies that differ from those of the *Orthopoxvirus* genus.

The variety of immune evasion molecules observed in MCV may result from its unique tissue tropisms and the specific diseases it causes. Unlike other poxviruses, MCV exclusively infects humans [6]. Although MCV lesions usually do not cause pain, they can be itchy or irritating. Scratching or picking at them may spread the infection to other areas, lead to bacterial infections, and result in scarring [7]. The disease is endemic, with higher infection rates observed in communities affected by overcrowding, poor sanitation, and poverty, which facilitate viral transmission. Globally, MCV prevalence ranges from 2% to 8% [8]. Particularly as a sexually transmitted infection that is often connected to HIV co-infection, MCV has become more common over the past 30 years. Research indicates that 5%–20% of HIV patients are impacted by MCV [9].

Since MC's symptoms are mild, and its effects are rare, people tend to think it happens less often than it really does. It is challenging to make reliable assays for seroprevalence studies because it is not simple to grow MCV in a lab [10]. Seroprevalence studies that used enzyme-linked immunosorbent assay (ELISA) had different results, mainly because of the viral antigen that was selected [11–13].

The most accurate way to identify MCV is to use tests based on the polymerase chain reaction (PCR). One of the advantages of molecular diagnostics is that they can determine the exact type of *Molluscum* strain causing the infection [14, 15]. In the past, studies in Diyala Province that used PCR to find genes have shown different rates of detection. For example, Al-Azawi [16] found that 45.1% of people between the ages of 30 and 41 had MCV, and Ahmed [17] found that 78.4% of head and neck samples had MCV. Mohammed's [18] study found that 53.3% of children aged 1–10 years were able to be detected. Hence, we aimed in this study to build on these findings by investigating MCV in skin tissue samples from people of different ages and targeting specific genes.

In this work, we targeted the *MC021L* gene in 101 clinically suspected cases. Then, molecular screening of MCV was performed in Diyala Province, Iraq. Using phylogenetic analysis, sequencing, and PCR amplification, the *MC021L* gene encodes a viral envelope protein homologous to vaccinia virus (VACV) F13L (40% amino acid similarity). In VACV, F13 and B5 proteins are essential for producing wrapped virions; deletions in their genes (F13L/B5R) abolish enveloped forms and impair cell-to-cell transmission [19]. *MC021L*'s functional conservation suggests it may similarly govern MCV spread and represents a potential target for antipoxviral drugs [15].

## 2. Experimental Part

### 2.1. Patients and Methods

Diyala Province served as the site of this cross-sectional study from October 2020 to April 2021. Ethical compliance was ensured by official approvals from Baquba Teaching Hospital in Diyala to collect samples, and verbal consent was obtained from each participant. The participants' details regarding age, gender, education, residence, previous infection, concomitant diseases, MCV duration, MCV development, MCV site, MCV nature, and MCV size are listed in Tables S1–S11, respectively. Furthermore, Figure S1 shows the concomitant diseases in MCV-positive patients. Patients with a clinical suspicion of MC infection provided 101 skin tissue samples. With ages ranging from 5 months to 60 years and illness durations from 1 week to 1 year, participants were drawn from the Dermatology Clinic of Baquba Teaching Hospital and several primary healthcare centers. A customized questionnaire was created to collect sociodemographic and clinical data. A qualified dermatologist obtained cutaneous lesions (Mollusca) from each patient, subsequently placing them in sterile tubes containing 2 mL of phosphate-buffered saline (pH 7.2) and storing them at −30°C before DNA extraction. This process employed the ReliaPrep Blood gDNA Miniprep System, supplemented with agarose, ethidium bromide solution (10 mg/mL), GoTaq Green Master Mix, nuclease-free water, and TAE 40X. Amplification of target genes was carried out via PCR using the primers specified in Table 1. The primers for the *MC021L* gene were selected based on their validation in the study by Taghinezhad et al. [15], which demonstrated their effectiveness for MCV detection. These primers target a conserved 979 bp region of the *MC021L* gene that contains the A27451G polymorphism, a known marker for distinguishing between MCV-1 and MCV-2 subtypes.

In the PCR procedure, 5 μL of extracted DNA was mixed with 10 μL of master mix, 1 μL each of forward and reverse primers, and 3 μL of nuclease-free water in a PCR tube, resulting in a total volume of 20 μL. The mixture was then placed into a PCR system (Bio-Rad, USA), and the program was set as 95°C initial denaturation for 5 min, followed by 40 cycles of 95 denaturation for 30 s, annealing at 55°C for 30 s and extension at 72°C for 30 s, and final extension at 72°C for 7 min.

### 2.2. PCR Amplicons' Nucleic Acids Sequencing

The PCR amplicons were sequenced in the forward direction by Macrogen Inc., Geumcheon, Seoul, South Korea, following their standard protocols. Only high-quality chromatograms from Applied Biosystems (ABI) sequence files were analyzed to rule out any potential PCR or sequencing errors. The observed nucleic acid sequences of local samples were compared with reference sequences to identify the exact positions and characteristics of the PCR fragments.

## 3. Results and Discussion

### 3.1. Region Detection of the MCV by Specific Primers

The *MC021L* gene was amplified in the samples employing specific primers and the PCR method; the reaction products showed DNA confirmation by agarose gel electrophoresis. The *MC021L* gene was selected for amplification for the 979 bp measurement. Using the SNP A27451G locus of the *MC021L* gene, MCV-positive samples were genotyped [15]. This gene is essential for MCV genotyping as it encodes a critical viral structural protein [20]. It shares homology with VACV F13L [21], which encodes the p37 membrane protein (a major component of the extracellular virion envelope that mediates viral egress and spread). The outer membrane is primarily derived from the trans-Golgi or endosomal compartments surrounding intracellular mature virions [22].

**Figure 1:**
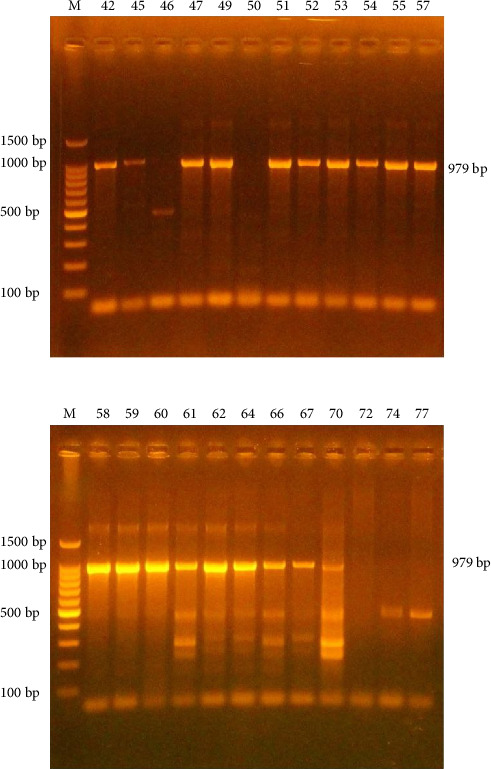
Amplification of the *MC021L* primer in human samples, separated on a 1.5% agarose gel and stained with ethidium bromide (EtBr). M represents the 100 bp ladder marker. Positive 979 bp PCR products are visible in Lanes 58–70 and 42–57, except 46 and 50 were negative.

Housed in the Golgi network, the F13L gene product p37K is a nonglycosylated polypeptide of 372 amino acids with cysteine residues at Positions 185 and 186 [23]. P37, the most abundant protein in the VACV envelope, is essential for envelope assembly, viral release, and transmission [19]. The PCR findings indicated a product size of 979 base pairs. Among the 101 samples examined, 19 (18.8%) were positive as illustrated in Table 2 and Figure 1, the remaining samples tested negative for MCV.

Although nonspecific bands were observed in some samples, nonspecific primer binding may occur due to several factors, including repetitive sequences in the DNA template, mismatched primer–template interactions, extreme G-C content, or partial annealing that leaves the 5′ end of the primer unbound.

This study's findings highly contrast with those of Taghinezhad et al. [15] in Iran, who documented a detection rate of 7.7% (114 out of 1470 samples) for the identical 979 bp gene. The observed variation may stem from mutations in the gene region, the emergence of a novel strain, discrepancies in experimental methodologies, genotypic variations, or environmental and climatic factors influencing viral isolation.

### 3.2. Nucleotide Sequence of the *MC021L* Gene in Amplified Pieces

Only positives with high-quality PCR bands were isolated from different sites of the patient's body. These samples represented diverse anatomical sites of infection, including facial, leg, abdominal, auricular, genital, and hand lesions, providing comprehensive coverage of clinical manifestations. Within this locus, six samples (only positives with specific bands at 979 bp) were included in the present study. These samples were screened to amplify the *MC021L* gene sequences of MCV. Thus, the variation of the *MC021L* gene can be used for MCV genotyping due to its possible ability to adapt to variable genetic diversity, as seen in different viral types.

The result of sequencing the PCR products from the selected samples was edited, aligned, and analyzed in conjunction with corresponding reference sequences utilizing BioEdit Sequence Alignment Editor Software Version 7.1 (DNASTAR, Madison, WI, USA). Variations detected in each sequenced sample were documented according to their position within the PCR amplicons and their corresponding locations in the reference genome. All identified nucleotide variants in the viral sequences were annotated utilizing SnapGene Viewer Version 4.0.4 (https://www.snapgene.com).

**Figure 2:**
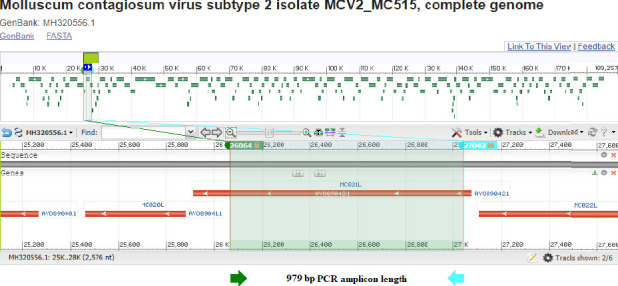
Location of the 979 bp amplicon within the *MC021L* gene segment of the MCV genome (GenBank accession no. MH320556.1). The green arrow marks the amplicon start site, and the cyan arrow indicates its end.

Reflecting the virus's capacity to evolve into different genetic alterations across several strains, the *MC021L* gene's genetic variation makes it a possible tool for viral genotyping. NCBI BLASTn analysis verified the identity of the PCR amplicons by sequencing results [24]. According to the NCBI BLASTn engine, the 979 bp amplicons showed 94.7% sequence similarity to the reference target sequences. The precise locations and features of the PCR fragments were defined by matching the discovered nucleic acid sequences of the specimens with the reference sequences (GenBank accession no. MH320556.1). The NCBI server was applied to identify the total length of the targeted locus; start and end locations were confirmed, as presented in Figure 2.

Following the alignment of 979 bp amplicon sequences with the MCV genome, the sequence details were examined, concentrating on the locations of the reverse and forward primers utilized for amplification, as outlined in Table 3.

**Figure 3:**
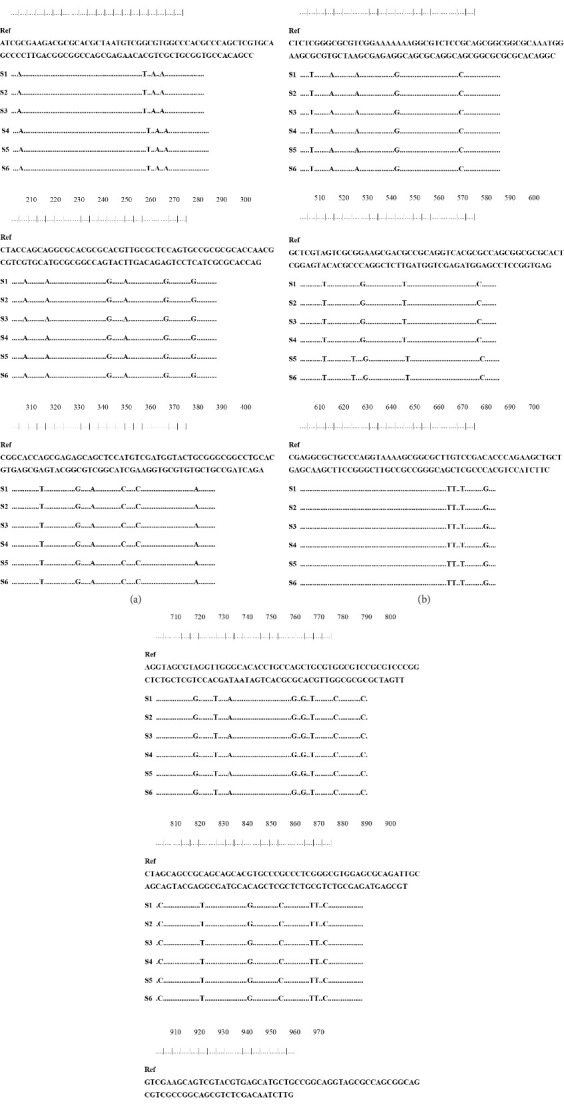
Nucleic acid alignment sequence of six samples with the reference sequence for the 979 bp amplicon of the *MC021L* gene. “Ref” denotes the NCBI reference sequence, while “S” followed by a number identifies each sample.

The 979 bp samples alignment showed 52 mutations compared to the most analogous reference nucleic acid sequences, as depicted in Figure 3.

**Figure 4:**
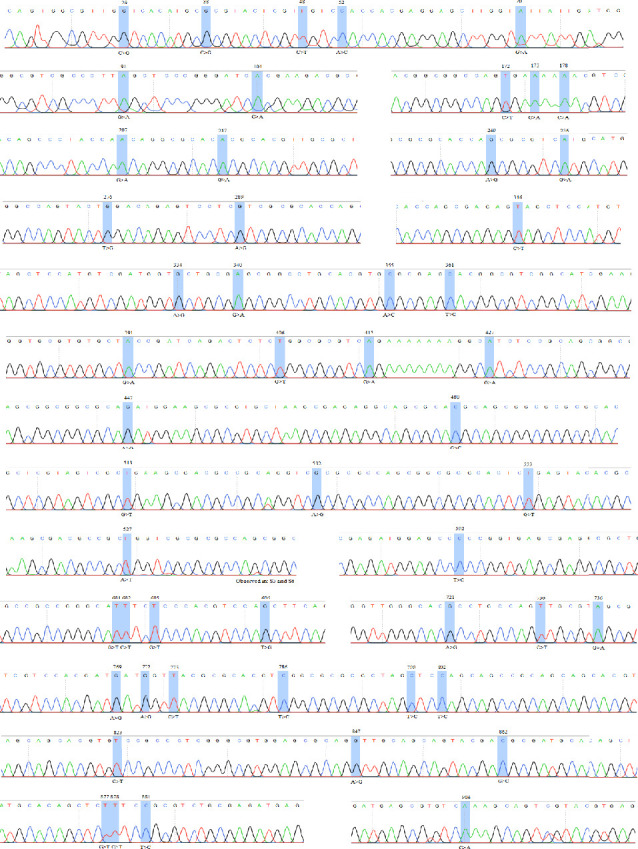
Chromatogram of the *Molluscum contagiosum* virus samples analyzed in this study. The clear nucleotide peaks demonstrate the rigorous contamination-free protocols used to validate each variant. “S” represents the sample codes, and all changes were consistently found in samples S1–S6 unless noted otherwise.

The discrepancies in the nucleic acid sequences of the examined samples were absent in the reference sequences. The sequencing chromatograms and their annotations were meticulously examined and recorded to guarantee the precision of the various variations. The chromatograms were arranged based on their locations within the PCR amplicons, as presented in Figure 4. Each variant was validated in its original chromatogram, and the absence of technical errors was likewise verified.

**Figure 5:**
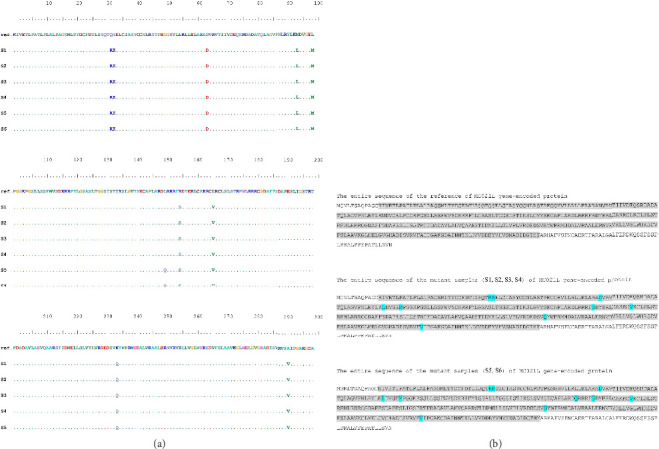
Alignment of amino acid residues showing identified substitutions in the *MC021L*-encoded palmitoylated EEV membrane glycoprotein from *Molluscum contagiosum* virus samples. (a) Substitutions mapped within the 979 bp amplified region. (b) Substitutions are displayed across the full-length protein sequence. Gray shading denotes the amplified region, while cyan highlights indicate amino acid substitutions.

The observed mutations were analyzed for their potential impact on the encoded *MC021L* protein. The nucleic acid sequences were translated into amino acid sequences using the ExPASy translate tool. Of the 52 identified nucleic acid substitutions, amino acid alignment with reference sequences (GenBank acc. no. MH320556.1) revealed that certain mutations were silent, whereas others resulted in missense effects (Figure 5(a)). The variations were charted throughout the entire protein sequence, indicating that most of the nucleic acid alterations were silent. The silent mutations included p.F37 = , p.A51 = , p.N56 = , p.G64 = , p.L71 = , p.E72 = , p.V80 = , p.T81 = , p.I82 = , p.A93 = , p.Q95 = , p.G98 = , p.E111 = , p.G141 = , p.S154 = , p.R161 = , p.L190 = , p.D196 = , p.S200 = , p.P203 = , p.G208 = , p.V218 = , p.A220 = , p.A225 = , p.S227 = , p.L233 = , p.D242 = , p.H253 = , p.L256 = , p.R266 = , p.L270 = , p.V279 = , p.F280 = , p.S281 = , p.A308 = , p.D322 = , and p.V321= (Figure 5(b)).

Conversely, certain nucleic acid variations led to missense mutations in the *MC021L* protein, including p.326V > A, p.329S > T, p.304A > V, p.323D > N, p.179L > V, p.247K > Q, p.163L > Q, p.168R > S, p.107M > L, p.112L > M, p.46S > K, p.45Q > R, and p.77N > D. The amino acid alterations in the *MC021L*-encoded glycoprotein are likely to induce substantial structural and functional changes, which potentially affect its interaction with the host's immune system. These alterations may indicate the virus's adaptation to circumvent pharmacological agents aimed at its glycoprotein [25, 26]. The presence of considerable amino acid substitutions in the MC021L gene-encoded glycoprotein of MC entails that such an altered protein may exhibit a considerable difference from its normal counterpart. Thus, due to these amino acid changes, the altered glycoprotein would participate in different metabolic pathways than those found in the wild-type glycoprotein.

The sequenced 979 bp fragments revealed the exact positions and annotations of the nucleotide substitution mutations, which were aligned with NCBI reference sequences. These mutations are presented in Table S12 (silent mutations) and Table S13 (missense mutations).

### 3.3. Phylogenetic Tree

**Figure 6:**
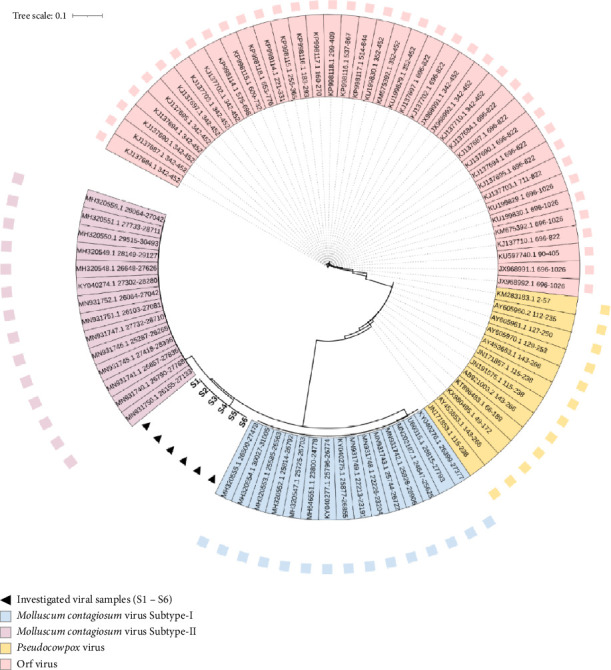
Comprehensive cladogram showing the phylogenetic relationships of genetic variants in the *MC021L* fragment from six local *Molluscum contagiosum* virus samples. The black-colored triangle refers to the analyzed MCV variants. All the stated numbers referred to the GenBank accession number of each referring species. The number “0.1” at the top portion of the tree refers to the degree of scale range among the comprehensive tree-categorized organisms. Colored circles at branches correspond to bootstrap values > 95%. The taxonomy of species (class) is represented by a colored strip. The letter “S#” indicates the studied samples' code.

This study constructed a detailed phylogenetic tree based on nucleotide variations detected within the amplified 979 bp segment of the *MC021L* amplicons. The tree included samples S1, S2, S3, S4, S5, and S6, as well as supplementary MCV sequences. Two separate parapoxviruses, *Pseudocowpox* virus and Orf virus, were included to delineate an outgroup within the phylogenetic framework. The pathogenic outgroup viruses displayed nucleic acid sequences closely resembling those of the analyzed MC samples. In the final tree, eighty-six nucleic acid sequences were aligned. The constructed cladogram placed the analyzed samples within a singular clade in the MCV sequence region, as illustrated in Figure 6.

**Figure 7:**
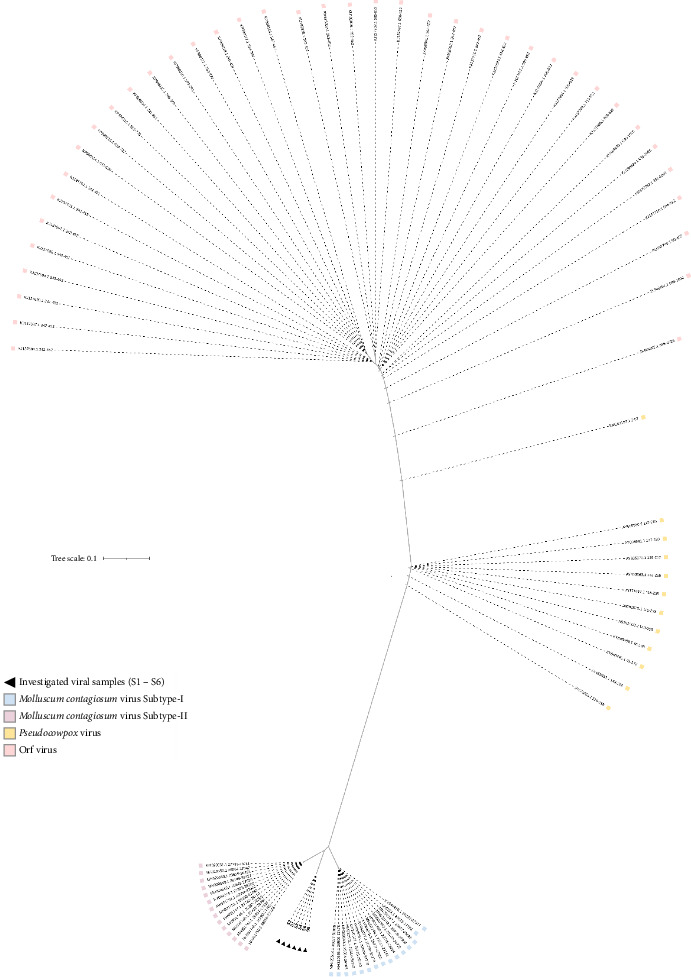
An unrooted phylogenetic tree that depicts the genetic variations of the *MC021L* fragment from six local *Molluscum contagiosum* virus samples. The analyzed viral variants are marked with a black triangle, and the numbers correspond to the GenBank accession numbers of reference species. The scale bar (“0.1”) at the top represents the genetic distance among the organisms, with the “S#” labels indicating the studied samples.

Our study's most striking result is the viral isolates' clear placement in the tree of life, between MC Subtypes-I and -II. The unrooted version of the evolutionary tree made this unusual location quite evident. Neither Subtype-I nor Subtype-II could be applied to these sequences, as demonstrated by the unrooted tree. Rather, these sequences stand out from all other subtypes that have been previously identified, according to the neighbor-joining cladograms (Figure 7).

The extensive mutations identified in these samples resulted in the grouping of all S1–S6 samples into a single clade. No other MCV isolates were placed within this clade, highlighting the unique genetic composition of these viral strains. Positioned between the Subtype-I and Subtype-II clades, the S1–S6 clade exhibits notable nucleic acid variations, which represent significant polymorphisms within the primary MCV lineage. The consistent clustering of these viral samples suggests a distinct pattern in their phylogenetic distribution. The marked divergence of the S1–S6 clade is due to 52 specific mutations that are absent in the viral sequences of the other couple subtypes. This divergence further emphasizes the genetic distinctiveness of the S1–S6 clade and its unique position within the phylogenetic structure of the MCV.

By demonstrating a remarkable frequency of nucleic acid variations in relation to known subtypes, the phylogenetic analysis of this work has validated the sequencing method. This strongly implies that the S1–S6 sequences are from a putative new clade of MC. The results are supported by the *MC021L* gene sequences, which display notable amino acid alterations in the glycoprotein encoded by *MC021L*. These variations could create structural and functional deviations from the wild-type protein that could affect its metabolic interactions. The information confirms the S1, S2, S3, S4, S5, and S6 sequence categorization as a putative new clade of the MCV.

The phylogenetic tree produced using the *MC021L* gene is a consistent tool for exactly identifying *MC021L* samples, depending on this genetic marker. Our work demonstrates how effectively *MC021L*-specific primers can identify evolutionary sites and characterize MCV, where all tested samples fit under one phylogenetic group based on the nucleic acid variations observed in the phylogenetic tree. These variations appear to produce high evolutionary changes in the examined viral samples under the MC lineage. The unusual location of these samples most likely results from the adaptive character of the *MC021L* fragment, which shows nucleic acid changes in reaction to host immune system interactions [27].

## 4. Conclusions

The most intriguing feature of our viral isolates is their unique phylogenetic position between MCV Subtypes-I and -II. The unrooted phylogenetic tree made it clear that the isolates did not group with either known subtype. Because of the many mutations, all of the S1–S6 samples made a separate clade. This clade contained only our viral samples and did not include any MCV isolates that had been previously reported. This suggests that these sequences have genetic differences that are not found in other MCV isolates; in fact, these changes in nucleic acids are important polymorphisms compared to the main MCV clade. Putting all the samples together into one clade suggests that they all have the same phylogenetic distribution pattern. The S1–S6 clade is likely different because it has 52 mutations that are not found in Subtypes-I and -II. The sequencing results were even more reliable because the phylogenetic tree explained a lot of the nucleotide changes compared to the known subtypes. The tree shows that all of the samples are in the same phylogenetic position because they all have the same nucleotide substitutions. These mutations could represent a remarkable shift in evolution, affecting where these isolates fit within the MCV phylogeny. The *MC021L* fragment may have changed its nucleotides in response to host immune pressure, which could explain its unique phylogenetic position. The changes in the *MC021L* sequence detected in the S1–S6 samples suggest that the virus has evolved a mechanism to evade the host's immune system. These notable genetic changes need to be further investigated to see if they mean that this is a new MCV subtype.

## Figures and Tables

**Table 1:** Forward and reverse primers.

Primer	Sequence	Anneal. temp. (°C)	Product size (bp)	Reference
*MC021L*-F	5′-CAAGATTGTAGAGACGCTGC-3′	55	979	[15]
*MC021L*-R	5′-GTAGTGCGTGCCGTCCATGT-3′			

**Table 2:** The rates of *MC021L* gene detection in the investigated samples.

Gene		No.	%
*MC021L* gene (979) bp	Negative	82	81.2
	Positive	19	18.8
	Total	101	100

**Table 3:** Length and position of the 979 bp PCR amplicon targeting a segment of the *MC021L* gene in the MCV genome (GenBank accession no. MH320556.1).

Amplicon	Reference locus sequences (5′-3′)	Length
Nucleic acid sequences within the viral *MC021L* gene	**^∗^GTAGTGCGTGCCGTCCATGT**CGGCGCTGGTCACATGCACGTACTCGTCGTCCACCACGAGGAGCTTGGTGTTATTGACGGCGTCGCCCTTAGCTCCCGGGATCGCGAAGACGCGCACGCTAATGTCGGCGTGGCCCACGCCCAGCTCGTGCAGCCCCTTGACGGCGGCCAGCGAGAACACGTCGCTGCGGTGCCACAGCCCTACCAGCAGGCGCACGCGCACGTTGCGTTCCAGTGCCGCGCGCACCAACGCGTCGTGCATGCGCGGCCAGTACTGTACAGAGTCCTCGTCGCGCACCAGTGGCACCAGCGAGAGTAGCTCCATGTCGATAGTACTGCGAGCGGCCTGCACGTGCGCGAGCACAGCGTCGGCATCAAAGGTGCGTGTGCTGCCGATAAGACTTTCCGGCGCGTCGGAAAAGAAGGCGTTTTCGCAGTGGCGGCGCAAATGGAAGCGCGTGCTAAGCGAGAGGCAGCGCACGCAGCGGCGCGCGCACAGGCGCTCGTAGTCGCGGAAGCGGCGACGCAGGTCACGCGCCAGCGGCTCGCACTCGGAGTACACGCCCAGGCTCTTGATGGTCGAAATGGAGCCCCCGGTAAGCGAGGCGCTGCCCAGGTAAAAGCGGCGCTTGTCCGATACCCAGAAGCTGCTGAGCAAGCTTCCGGGCTTGCCGCCGGGCAGTTCGCCCACGTCCAGCTTCAGGTAGCGTAGGTTGGGCACGCCCGCCAGCTGCGTGGCATCCGCGTCCCGGCTCTGCTCGTCCACGATAATCGTCACGCGCACGTCGGCGCGCGCTAGCTCCAGCAGCCGCAGCAGCACGTGCCCGCCCTCGGGCGTGGAGCGCAGATTGCAGCAGTAAGACGCAATGCACAGCTCGCGCTGCGTCTGCGAGATGAGCGTGTCAAAGCAGTCGTACGTGAGCATGCTGCCGGTAGGTAGCGCCAGCGGCAGCGTTGCCG**GCAGCGTCTCTACAATCTTG**^∗∗^	979 bp

*Note:* Forward and reverse primer positions are highlighted in bold.

^∗^Denotes the R primer sequences (set in a forward direction).

^∗∗^Denotes the F primer sequences (set in a reverse complement direction).

## Data Availability

The data that support the findings of this study are available from the corresponding author upon reasonable request.

## Supporting Information

Table S1. Age distribution of patients.

Table S2. Gender distribution of patients.

Table S3. Distribution of educational levels of patients in this study.

Table S4. Residence distribution of patients.

Table S5. Previous infection with MCV in patients.

Table S6. Concomitant diseases of MCV patients.

Table S7. Duration of the MCV lesion in patients.

Table S8. Season of MCV lesion development.

Table S9. Distribution of the site of the MCV lesion.

Table S10. Nature of MCV lesions.

Table S11. Distribution of the size of MCV lesions.

Table S12. Observed SNP patterns (silent mutation) in the 979 bp amplicons of the *MC021L* gene, aligned with NCBI reference sequences (GenBank accession no. MH320556.1). “S” with a number identifies the viral sample analyzed.

Table S13. Observed SNP patterns (missense mutation) in the 979 bp amplicons of the *MC021L* gene, aligned with NCBI reference sequences (GenBank accession no. MH320556.1). “S” with a number identifies the viral sample analyzed.

Figure S1. Concomitant diseases in MCV-positive patients.

## References

[1] Leung A. K., Barankin B., Hon K. L. (2017). Molluscum Contagiosum: An Update. *Recent Patents on Inflammation & Allergy Drug Discovery*.

[2] Chen X., Anstey A. V., Bugert J. J. (2013). Molluscum Contagiosum Virus Infection. *The Lancet Infectious Diseases*.

[3] Trčko K., Hošnjak L., Kušar B. (2018). Clinical, Histopathological, and Virological Evaluation of 203 Patients With a Clinical Diagnosis of Molluscum Contagiosum. *Open Forum Infectious Diseases*.

[4] Aldabagh B., Ly M. N., Hessel A. B., Usmani A. S. (2010). Molluscum Contagiosum Involving an Epidermoid Cyst With Xanthogranuloma‐Like Reaction in an Hiv‐Infected Patient. *Journal of Cutaneous Pathology*.

[5] Luke J. D., Silverberg N. B. (2010). Vertically Transmitted Molluscum Contagiosum Infection. *Pediatrics*.

[6] Shisler J. L. (2015). Immune Evasion Strategies of Molluscum Contagiosum Virus. *Advances in Virus Research*.

[7] Basta-Juzbašić A., Čeović R. (2014). Chancroid, Lymphogranuloma Venereum, Granuloma Inguinale, Genital Herpes simplex Infection, and Molluscum Contagiosum. *Clinics in Dermatology*.

[8] Maytham M., Abbas M. Y. (2012). Atypical Clinical Presentation of Molluscum. *Al-Kindy College Medical Journal*.

[9] Gatea M. A., Humoud M. N., Al-Hmudi H. A. (2019). Molecular Detection of Molluscum Contagiosum Virus (MCV) from Patients of Basra Province/Iraq. *Scientific Journal of Medical Research*.

[10] Buller R. M., Burnett J., Chen W., Kreider J. (1995). Replication of Molluscum Contagiosum Virus. *Virology*.

[11] Konya J., Thompson C. H. (1999). Molluscum Contagiosum Virus: Antibody Responses in Persons With Clinical Lesions and Seroepidemiology in a Representative Australian Population. *The Journal of Infectious Diseases*.

[12] Watanabe T., Nakamura K., Wakugawa M. (2000). Antibodies to Molluscum Contagiosum Virus in the General Population and Susceptible Patients. *Archives of Dermatology*.

[13] Sherwani S., Farleigh L., Agarwal N. (2014). Seroprevalence of Molluscum Contagiosum Virus in German and UK Populations. *PLoS One*.

[14] Trama J. P., Adelson M. E., Mordechai E. (2007). Identification and Genotyping of Molluscum Contagiosum Virus from Genital Swab Samples by real-time PCR and Pyrosequencing. *Journal of Clinical Virology*.

[15] Taghinezhad-S S., Mohseni A. H., Keyvani H., Ghobadi N. (2018). Molecular Screening and Single Nucleotide Polymorphism Typing of Molluscum Contagiosum Virus (MCV) from Genital Specimens, Between 2012 and 2015. *Iranian Biomedical Journal*.

[16] Al-Azawi M. K. (2013). Polymerase Chain Reaction for Detection and Genotyping of Molluscum Contagiosum Virus in Diyala Province. *Journal of Medicine*.

[17] Ahmed R. (2013). *Polymerase Chain Reaction for Detection and Genotyping of Molluscum Contagiosum Virus in Diyala Province*.

[18] Mohammed S. (2020). Molecular Study of Molluscum Contagiosum Virus in Diyala Province.

[19] Bárcena J., Lorenzo M. M., Sánchez-Puig J. M., Blasco R. (2000). Sequence and Analysis of a Swinepox Virus Homologue of the Vaccinia Virus Major Envelope Protein P37 (F13L). *Journal of General Virology*.

[20] López-Bueno A., Parras-Moltó M., López-Barrantes O., Belda S., Alejo A. (2017). Recombination Events and Variability Among full-length Genomes of co-circulating Molluscum Contagiosum Virus Subtypes 1 and 2. *Journal of General Virology*.

[21] Senkevich T. G., Koonin E. V., Bugert J. J., Darai G., Moss B. (1997). The Genome of Molluscum Contagiosum Virus: Analysis and Comparison With Other Poxviruses. *Virology*.

[22] Husain M., Weisberg A., Moss B. (2003). Topology of epitope-tagged F13L Protein, a Major Membrane Component of Extracellular Vaccinia Virions. *Virology*.

[23] Grosenbach D. W., Ulaeto D. O., Hruby D. E. (1997). Palmitylation of the Vaccinia Virus 37-kDa Major Envelope Antigen: Identification of a Conserved Acceptor Motif and Biological Relevance. *Journal of Biological Chemistry*.

[24] Zhang Z., Schwartz S., Wagner L., Miller W. (2000). A Greedy Algorithm for Aligning DNA Sequences. *Journal of Computational Biology*.

[25] Sarhan S. R., Hashim H. O., Al-Shuhaib M. B. S. (2020). The Gly152Val Mutation Possibly Confers Resistance to beta-lactam Antibiotics in Ovine *Staphylococcus aureus* Isolates. *Open Veterinary Journal*.

[26] Topalis D., Gillemot S., Snoeck R., Andrei G. (2016). Distribution and Effects of Amino Acid Changes in drug-resistant Α and Β Herpesviruses DNA Polymerase. *Nucleic Acids Research*.

[27] Al-Malkey M. K., Al-Obaidi M. J., Mohammed S. W., Nayyef H. J., Jabbar F., Al-Deeri M. M. (2019). Pearl Skin Disease Comprehension Among University of Baghdad Students. *Age*.

