# Supplementary material for: Molecular Characterization of Molluscum Contagiosum Virus: Identification of a Putative New Clade Through MC021L Gene Analysis in Diyala Province, Iraq
Source: Adv Virol. 2025 Aug 5;2025:1574406. doi: 10.1155/av/1574406 (PMC12343169; doi:10.1155/av/1574406)
Supplement: Supporting Information — Additional supporting information can be found online in the Supporting Information section. [file 1574406.f1.docx]

**Molecular Characterization of Molluscum Contagiosum Virus: Identification of a Putative New Clade through MC021L Gene Analysis in Diyala Province, Iraq**

Sameer M. Dheyab^1^, Zahraa. J. Jameel^2,*^, Ansam Dawod Salman^1^, Mohammed Kadhom^2^

^1^ Department of Biology, College of Science, Diyala University, Baqubah, Diyala, 32001, Iraq

^2^ Department of Environmental Health Science, College of Energy and Environmental Science, Al-Karkh University of Science, Baghdad, 10081, Iraq

*Corresponding Author Email: [dr.zahraa.j@kus.edu.iq](mailto:dr.zahraa.j@kus.edu.iq)

**S1- Details of Patients**

The participants’ details regarding age, gender, education, residence, previous infection, concomitant diseases, MCV duration, MCV development, MCV location, MCV nature, MCV frequency, and MCV size are listed in Tables S1-S11, respectively. Furthermore, Figure S1 shows the concomitant diseases in MCV-positive patients.

**Table S1. Age distribution of patients.**

| **Age (Years)** | **No.** | **%** | **P value** |
| --- | --- | --- | --- |
| <10 years | 56 | 55.4 | **0.0001*** |
| 10-19 | 8 | 7.9 |  |
| 20-29 | 11 | 10.9 |  |
| 30-39 | 10 | 9.9 |  |
| ≥ 40 years | 16 | 15.8 |  |
| Total | 101 | 100 |  |

*Significant difference between percentages using Pearson Chi-square test (χ2-test) at 0.05 level.

S1-2 Gender distribution

**Table S2. Gender distribution of patients.**

| **Gender** | **No.** | **%** | **P value** |
| --- | --- | --- | --- |
| Male | 53 | 52.5 | **0.619** |
| Female | 48 | 47.5 |  |
| Total | 101 | 100 |  |

*Significant difference between percentages using Pearson Chi-square test (χ2-test) at 0.05 level

**Table S3. Distribution of educational levels of patients in this study.**

| **Educational levels** | **No.** | **%** | **P value** |
| --- | --- | --- | --- |
| Illiterate | 49 | 49.5 | **0.0001*** |
| Primary | 21 | 21.2 |  |
| Secondary | 4 | 4.0 |  |
| Institute | 10 | 10.1 |  |
| College | 15 | 15.2 |  |

*Significant difference between percentages using Pearson Chi-square test (χ2-test) at 0.05 level.

**Table S4. Residence distribution of patients.**

| **Residence** | **No.** | **%** | **P value** |
| --- | --- | --- | --- |
| Urban | 34 | 33.7 | **0.001*** |
| Rural | 67 | 66.3 |  |
| Total | 101 | 100 |  |

*Significant difference between percentages using Pearson Chi-square test (χ2-test) at 0.05 level.

**Table S5. Previous infection with MCV of patients.**

| **Previous infection** | **No.** | **%** | **P value** |
| --- | --- | --- | --- |
| Yes | 20 | 19.8 | **0.0001*** |
| No | 81 | 80.2 |  |
| Total | 101 | 100 |  |

*Significant difference between percentages using Pearson Chi-square test (χ2-test) at 0.05 level

**Table S6. Concomitant diseases of MCV patients.**

| **Previous infection** | **No.** | **%** | **P value** |
| --- | --- | --- | --- |
| Yes | 55 | 54.5 | **0.371** |
| No | 46 | 45.5 |  |
| Total | 101 | 100 |  |

*Significant difference between percentages using Pearson Chi-square test (χ2-test) at 0.05 level.

**Table S7. Duration of the MCV lesion in patients.**

| Duration of infection (ms) | **No.** | **%** | **P value** |
| --- | --- | --- | --- |
| One month | 52 | 51.5 | 0.0001* |
| Two months | 37 | 36.6 |  |
| Three months or more | 12 | 11.9 |  |
| Mean ± SD (Range) | 5.9±4.4 months (1-28) months | | |

*Significant difference between percentages using Pearson Chi-square test (χ2-test) at 0.05 level.

**Table S8. Season of MCV lesion development.**

| **Season** | **No.** | **%** | **P value** |
| --- | --- | --- | --- |
| Winter | 39 | 38.6 | **0.022*** |
| Spring | 62 | 61.4 |  |
| Total | 101 | 100 |  |

*Significant difference between percentages using Pearson Chi-square test (χ2-test) at 0.05 level.

**Table S9. Distribution of the site of the MCV lesion.**

| **Site of lesion** | **No.** | **%** | **P value** |
| --- | --- | --- | --- |
| Face, nose, ear, eye, and lips | 60 | 59.4 | **0.0001*** |
| Head and neck | 10 | 9.9 |  |
| Chest, back, and abdominal wall | 15 | 14.9 |  |
| Hand, armpit, leg, and feet | 11 | 10.9 |  |
| Anal region and genitalia | 5 | 5.0 |  |
| Total | 101 | 100 |  |

*Significant difference between percentages using Pearson Chi-square test (χ2-test) at 0.05 level.

**Table S10. Nature of MCV lesions.**

| **Season** | **No.** | **%** | **P value** |
| --- | --- | --- | --- |
| Single | 87 | 86.1 | **0.0001*** |
| Cluster | 14 | 13.9 |  |
| Total | 101 | 100 |  |

*Significant difference between percentages using Pearson Chi-square test (χ2-test) at 0.05 level.

**Table S11. Distribution of the size of MCV lesions.**

| **Size of lesion (mm)** | **No.** | **%** | **P value** |
| --- | --- | --- | --- |
| 1 | 8 | 7.9 | **0.0001*** |
| 2 | 52 | 51.5 |  |
| 3 | 1 | 1.0 |  |
| 4 | 15 | 14.9 |  |
| 5 | 18 | 17.8 |  |
| 6 | 7 | 6.9 |  |
| Total | 101 | 100 |  |
| Mean ± SD (Range) | 2.7 ± 1.1 (1-5) | | |

*Significant difference between percentages using Pearson Chi-square test (χ2-test) at 0.05 level.

**Figure S1. Concomitant diseases in MCV-positive patients**

**S2- SNP patterns**

**Table S12. Observed SNP patterns (silent mutation) in the 979 bp amplicons of the MC021L gene, aligned with NCBI reference sequences (GenBank accession no. MH320556.1). “S” with a number identifies the viral sample analyzed.**

| **Sample No.** | **Native** | **Allele** | **Position of nucleic acid in the PCR fragment** | **Position of nucleic acid in the reference genome** | **Position in the reference amino acid of the entire protein** | **Type of mutation** |
| --- | --- | --- | --- | --- | --- | --- |
| S1, S2, S3, S4, S5, S6 | A | C | 52 | 26115 | V321 | Silent (p.V321=) |
| S1, S2, S3, S4, S5, S6 | G | A | 70 | 26113 | D322 | Silent (p.D322=) |
| S1, S2, S3, S4, S5, S6 | G | A | 91 | 26154 | A308 | Silent (p.A308=) |
| S1, S2, S3, S4, S5, S6 | C | T | 172 | 26235 | S281 | Silent (p.S281=) |
| S1, S2, S3, S4, S5, S6 | G | A | 175 | 26238 | F280 | Silent (p.F280=) |
| S1, S2, S3, S4, S5, S6 | C | A | 178 | 26241 | V279 | Silent (p.V279=) |
| S1, S2, S3, S4, S5, S6 | G | A | 207 | 26270 | L270 | Silent (p.L270=) |
| S1, S2, S3, S4, S5, S6 | G | A | 217 | 26280 | R266 | Silent (p.R266=) |
| S1, S2, S3, S4, S5, S6 | A | G | 249 | 26312 | L256 | Silent (p.L256=) |
| S1, S2, S3, S4, S5, S6 | G | A | 256 | 26319 | H253 | Silent (p.H253=) |
| S1, S2, S3, S4, S5, S6 | A | G | 289 | 26352 | D242 | Silent (p.D242=) |
| S1, S2, S3, S4, S5, S6 | C | T | 316 | 26379 | L233 | Silent (p.L233=) |
| S1, S2, S3, S4, S5, S6 | A | G | 334 | 26397 | S227 | Silent (p.S227=) |
| S1, S2, S3, S4, S5, S6 | G | A | 340 | 26403 | A225 | Silent (p.A225=) |
| S1, S2, S3, S4, S5, S6 | A | C | 355 | 26418 | A220 | Silent (p.A220=) |
| S1, S2, S3, S4, S5, S6 | T | C | 361 | 26424 | V218 | Silent (p.V218=) |
| S1, S2, S3, S4, S5, S6 | G | A | 391 | 26454 | G208 | Silent (p.G208=) |
| S1, S2, S3, S4, S5, S6 | G | T | 406 | 26469 | P203 | Silent (p.P203=) |
| S1, S2, S3, S4, S5, S6 | G | A | 415 | 26478 | S200 | Silent (p.S200=) |
| S1, S2, S3, S4, S5, S6 | G | A | 427 | 26490 | D196 | Silent (p.D196=) |
| S1, S2, S3, S4, S5, S6 | A | G | 447 | 26510 | L190 | Silent (p.L190=) |
| S1, S2, S3, S4, S5, S6 | A | G | 532 | 26595 | R161 | Silent (p.R161=) |
| S1, S2, S3, S4, S5, S6 | G | T | 553 | 26616 | S154 | Silent (p.S154=) |
| S1, S2, S3, S4, S5, S6 | T | C | 592 | 26655 | G141 | Silent (p.G141=) |
| S1, S2, S3, S4, S5, S6 | C | T | 682 | 26745 | E111 | Silent (p.E111=) |
| S1, S2, S3, S4, S5, S6 | A | G | 721 | 26784 | G98 | Silent (p.G98=) |
| S1, S2, S3, S4, S5, S6 | C | T | 730 | 26793 | Q95 | Silent (p.Q95=) |
| S1, S2, S3, S4, S5, S6 | G | A | 736 | 26799 | A93 | Silent (p.A93=) |
| S1, S2, S3, S4, S5, S6 | A | G | 769 | 26832 | I82 | Silent (p.I82=) |
| S1, S2, S3, S4, S5, S6 | A | G | 772 | 26835 | T81 | Silent (p.T81=) |
| S1, S2, S3, S4, S5, S6 | C | T | 775 | 26838 | V80 | Silent (p.V80=) |
| S1, S2, S3, S4, S5, S6 | T | C | 799 | 26862 | E72 | Silent (p.E72=) |
| S1, S2, S3, S4, S5, S6 | T | C | 802 | 26865 | L71 | Silent (p.L71=) |
| S1, S2, S3, S4, S5, S6 | C | T | 823 | 26886 | G64 | Silent (p.G64=) |
| S1, S2, S3, S4, S5, S6 | A | G | 847 | 26910 | N56 | Silent (p.N56=) |
| S1, S2, S3, S4, S5, S6 | G | C | 862 | 26925 | A51 | Silent (p.A51=) |
| S1, S2, S3, S4, S5, S6 | G | A | 904 | 26967 | F37 | Silent (p.F37=) |

**Table S13. Observed SNP patterns (Missense mutation) in the 979 bp amplicons of the MC021L gene, aligned with NCBI reference sequences (GenBank accession no. MH320556.1). “S” with a number identifies the viral sample analyzed.**

| **Sample No.** | **Native** | **Allele** | **Position of nucleic acid in the PCR fragment** | **Position of nucleic acid in the reference genome** | **Position in the reference amino acid of the entire protein** | **Type of mutation** |
| --- | --- | --- | --- | --- | --- | --- |
| S1, S2, S3, S4, S5, S6 | C | G | 29 | 26092 | S329 | Missense (p.329S>T) |
| S1, S2, S3, S4, S5, S6 | C | G | 38 | 26101 | V326 | Missense (p.326V>A) |
| S1, S2, S3, S4, S5, S6 | C | T | 48 | 26111 | D323 | Missense (p.323D>N) |
| S1, S2, S3, S4, S5, S6 | G | A | 104 | 26167 | A304 | Missense (p.304A>V) |
| S1, S2, S3, S4, S5, S6 | T | G | 276 | 26339 | K247 | Missense (p.247K>Q) |
| S1, S2, S3, S4, S5, S6 | G | C | 480 | 26543 | L179 | Missense (p.179L>V) |
| S1, S2, S3, S4, S5, S6 | G | T | 513 | 26576 | R168 | Missense (p.168R>S) |
| S5, S6 | A | T | 527 | 26590 | L163 | Missense (p.163L>Q) |
| S1, S2, S3, S4, S5, S6 | G | T | 681 | 26744 | L112 | Missense (p.112L>M) |
| S1, S2, S3, S4, S5, S6 | T | G | 696 | 26759 | M107 | Missense (p.107M>L) |
| S1, S2, S3, S4, S5, S6 | T | C | 786 | 26849 | N77 | Missense (p.77N>D) |
| S1, S2, S3, S4, S5, S6 | G | T | 877 | 26940 | S46 | Missense (p.46S>K) |
| S1, S2, S3, S4, S5, S6 | C | T | 878 | 26941 | S46 | Missense (p.46S>K) |
| S1, S2, S3, S4, S5, S6 | T | C | 881 | 26944 | Q45 | Missense (p.45Q>R) |
